# Phosphoproteomic analysis reveals plant DNA damage signalling pathways with a functional role for histone H2AX phosphorylation in plant growth under genotoxic stress

**DOI:** 10.1111/tpj.14495

**Published:** 2019-09-10

**Authors:** Wanda M. Waterworth, Michael Wilson, Dapeng Wang, Thomas Nuhse, Stacey Warward, Julian Selley, Christopher E. West

**Affiliations:** ^1^ Centre for Plant Sciences University of Leeds Woodhouse Lane Leeds LS2 9JT UK; ^2^ Leeds Omics University of Leeds Leeds LS2 9JT UK; ^3^ Faculty of Life Sciences University of Manchester Oxford Road Manchester M13 9PT UK

**Keywords:** ATAXIA TELANGIECTASIA MUTATED (ATM), DNA damage response, DNA repair, phosphorylation, seed

## Abstract

DNA damage responses are crucial for plant growth under genotoxic stress. Accumulating evidence indicates that DNA damage responses differ between plant cell types. Here, quantitative shotgun phosphoproteomics provided high‐throughput analysis of the DNA damage response network in callus cells. MS analysis revealed a wide network of highly dynamic changes in the phosphoprotein profile of genotoxin‐treated cells, largely mediated by the ATAXIA TELANGIECTASIA MUTATED (ATM) protein kinase, representing candidate factors that modulate plant growth, development and DNA repair. A C‐terminal dual serine target motif unique to H2AX in the plant lineage showed 171‐fold phosphorylation that was absent in *atm* mutant lines. The physiological significance of post‐translational DNA damage signalling to plant growth and survival was demonstrated using reverse genetics and complementation studies of *h2ax* mutants, establishing the functional role of ATM‐mediated histone modification in plant growth under genotoxic stress. Our findings demonstrate the complexity and functional significance of post‐translational DNA damage signalling responses in plants and establish the requirement of H2AX phosphorylation for plant survival under genotoxic stress.

## Significance Statement

This work demonstrates the central role for ATM in DNA damage signalling and establishes the functional requirement for H2AX phosphorylation in plant resistance to genotoxic stress. These results revealed the physiological significance of ATM‐dependent post‐translational protein modification in the plant DNA damage response in mitigating the deleterious cellular consequences of genome damage.

## Introduction

DNA damage, resulting from exposure to environmental stresses, including UV‐B and heavy metal soil pollutants, inhibits plant growth, threatens cellular survival and can result in the transmission of deleterious mutations to the next generation (Britt, 1996). In addition, damage to the genome arises from by‐products of cellular metabolism, in particular reaction oxygen species (ROS), and errors incurred during DNA replication and transcription. Increased exposure to environmental stresses causes greatly enhanced somatic recombination frequencies, genome instability and heritable mutations (Kovalchuk *et al*., 2000; Ries *et al*., 2000). A particularly cytotoxic and mutagenic form of DNA damage are DNA double‐stranded breaks (DSBs), which can lead to loss of large fragments of chromosomes if unrepaired. Consequently, plants, as other eukaryotes, have evolved powerful and complex DSB repair and response mechanisms which include delay of cell‐cycle progression, activation of DNA repair factors, endoreduplication and programmed cell death. In eukaryotes, DSB repair is mediated by either non‐homologous end‐joining (NHEJ) or homologous recombination (HR) pathways. Cellular responses to DNA damage are orchestrated by the phosphoinositide‐3‐kinase‐related protein kinases (PIKKs) ATAXIA TELANGIECTASIA MUTATED (ATM) and ATM AND RAD3‐RELATED (ATR). ATM is activated by chromosomal breaks (Culligan and Britt, 2008), whereas ATR responds to replication defects and is activated by regions of single‐stranded DNA including collapsed DNA replication forks and DSB repair intermediates (Culligan *et al*., 2004; Cools *et al*., 2011). In plants, both ATM and ATR activate programmed cell death and the switch to endocycles in response to genotoxins, whereas ATM mediates transcriptional responses to DNA damage with little contribution from ATR (Friesner *et al*., 2005; Culligan *et al*., 2006; Fulcher and Sablowski, 2009; Furukawa *et al*., 2010; Adachi *et al*., 2011). In mammals, ATM orchestrates the cellular response to DNA damage by phosphorylation of hundreds of substrates including repair factors and signalling proteins that activate cell‐cycle checkpoints or activate apoptosis (Matsuoka *et al*., 2007). Notably, plants lack clear homologues of many of these downstream factors, including p53, the effector kinases CHK1, CHK2, and apoptotic factors, indicating that signalling downstream of ATM and ATR may have evolved independently in the plant lineage (Hu *et al*., 2016). Some functionality of p53 is performed by the SUPPRESSOR OF GAMMA 1 (SOG1) transcription factor, which is required for the transcriptional DNA damage response, programmed cell death, endoreduplication and cell‐cycle arrest (Yoshiyama *et al*., 2009; Yoshiyama, 2015).

A conserved eukaryotic response to genotoxic stress is the ATM and ATR dependent C‐terminal phosphorylation of the histone variant H2AX (Downs *et al*., 2000; Turinetto and Giachino, 2015). H2AX phosphorylation (γ‐H2AX) is a very early event in the DNA damage response, occurring within minutes of DSB induction in mammals, and functions to recruit repair factors to the break site where it promotes repair of chromosomal breaks (Paull *et al*., 2000). H2AX phosphorylation takes place on a PIKK consensus ‘SQ’ motif in the C‐terminal tail, corresponding to S139 in Arabidopsis. DNA damage‐induced phosphorylation was abolished in an *atm atr* double mutant (Friesner *et al*., 2005), and phosphoproteomic analysis supported the conclusion that in Arabidopsis these two kinases are solely responsible for phosphorylating this residue in response to genotoxic stresses (Roitinger *et al*., 2015). Arabidopsis has two H2AX isoforms termed H2AXA (AT1G08880) and H2AXB (AT1G54690) that differ only in two residues in their primary structure. Previous studies used RNAi to silence *H2AX* expression, leading to a 50% reduction in *H2AXA* and *H2AXB* transcripts. The silenced lines displayed mild hypersensitivity to the genotoxins bleomycin and camptothecin (Lang *et al*., 2012). Further studies using T‐DNA mutant lines revealed altered DSB repair patterns in H2AX‐deficient plants (Qi *et al*., 2016). In mammals, C‐terminal phosphorylation of H2AX is important in promoting HR and loss of H2AX reduces DSB repair, leading to genomic instability, sterility and reduced repair activity (Celeste *et al*., 2002; Xie *et al*., 2004; Scully and Xie, 2013). The γ‐H2AX chromatin mark extends up to several megabases around the site of DNA damage and can be visualized using immunocytochemistry as discrete foci within the nucleus of genotoxin‐treated cells (Natale *et al*., 2017). In plants, as in animals, monitoring the appearance and disappearance of γ‐H2AX foci has provided a powerful mechanism for monitoring the presence of DSBs and progression of their repair (Charbonnel *et al*., 2011). However, given marked differences between H2AX activities in yeast and animals, and the lack of characterized γ‐H2AX‐interacting proteins in plants, the roles of DNA damage‐induced phosphorylation remain unclear, and its role in promoting plant genome stability in response to genotoxic stresses remains to be established.

While ATM and ATR activation is conserved between eukaryotes, there is significant divergence between eukaryotes in downstream DNA damage response pathways (Hu *et al*., 2016). Post‐translational signalling networks coordinate mammalian DNA damage responses, yet we know little of their functional roles in plant responses to genotoxic stress, beyond the requirement for phosphorylation of the transcription factor SOG1 by ATM (Yoshiyama *et al*., 2013). Phosphoproteomic analyses established extensive post‐translational DNA damage response networks in Arabidopsis and identified differences between wild‐type and *atm atr* double mutants (Roitinger *et al*., 2015). This study used gamma‐irradiated mature Arabidopsis plants, with proteins extracted from above ground tissues. However, maintenance of genome integrity is particularly important in plant meristematic cells to support plant growth and development and many plant DNA damage responses are specific to mitotically dividing cells (Hefner *et al*., 2006). In particular, plant stem cell initials display elevated levels of programmed cell death relative to the surrounding cell types in root and shoot tissues (Fulcher and Sablowski, 2009; Furukawa *et al*., 2010). Preferential transcriptional upregulation of many DNA repair factors is also observed in meristem tissues (Yadav *et al*., 2009; Da Ines *et al*., 2013). Moreover, in shoot meristems that give rise to the plant germline, mutations incurred in vegetative tissues can be transmitted to the next generation (Ries *et al*., 2000). Here, to advance our understanding of plant DNA damage responses, candidate downstream effectors of ATM phosphorylation were identified in callus tissue using quantitative shotgun phosphoproteomics. Comparative phosphoproteomic analysis of wild‐type and *atm‐3* mutants, with and without genotoxin treatment, revealed altered abundance of 225 phosphorylated peptides in response to DNA damage, 68% of which were dependent on ATM including 10 sites that were S/TQ ATM and ATR consensus sequences. Identified peptides corresponded to proteins with candidate roles in DNA repair, transcription and development. The DNA damage responsive phosphosites of cells from callus tissue displayed very little similarity (2%) to the post‐translational DNA damage response of mature Arabidopsis plants (Roitinger *et al*., 2015). This is consistent with the identification of downstream components of DNA damage signalling in the present study, given the longer duration of genotoxin treatment, and may also reflect a high degree of differentiation between the DNA damage signalling networks in different cell and tissue types. Further analysis revealed the functional requirement for H2AX phosphorylation in plant resistance to genotoxic stress. These results establish the functional significance of ATM‐dependent protein phosphorylation in the plant DNA damage response, crucial in mitigating the deleterious cellular consequences of damage to the genome.

## Results

### Phosphoproteomic analysis of the DNA damage response

Rapid and sensitive responses to genotoxic stresses are crucial to safeguard the fidelity of genetic information, in particular in the actively dividing cell populations of plant meristems. Passage through the cell cycle in the presence of DNA damage can result in mutation, aneuploidy and cell death (Sancar *et al*., 2004). Here we used a shotgun proteomic approach to identify phosphopeptides that change in abundance in response to X‐irradiation (100 Gy over 100 min) in callus cell populations of wild‐type and *atm‐3* plants. Callus tissues have elevated transcription of cell‐cycle factors (Supporting Information Figure S1) and here displayed highly dynamic post‐translational signalling networks operative in response to genotoxic stress. Quantitative MS identified 2220 phosphopeptides and 4798 phosphorylation sites (Table S1). 225 phosphopeptides (180 proteins, 254 sites) were identified that displayed significant change in abundance upon X‐irradiation of wild‐type lines (Table S2), analyzing four independent replicates for each treatment and correcting for a false discoveries using limma in R (van Ooijen *et al*., 2017). Of the 254 X‐ray responsive phosphorylation sites identified here, only six were previously identified in the DNA damage signalling network of mature wild‐type plants (Table S2), highlighting the wide range of phosphoproteins induced upon genotoxin treatment. The lack of overlap between the post‐translational DNA damage signalling response with the previous work is likely to reflect the different plant tissues studied (mature plants at stage 5.10 versus callus tissue), as well as technical differences between the two analyses (Roitinger *et al*., 2015). Notably, peptides identified the present study are likely to include signalling components downstream of ATM, given the length of time tissues were irradiated (100 min in the present study, whereas Roitinger *et al*. (2015) irradiated for 4 min followed by 15 min recovery). Phosphorylation of several DNA repair factors was detected, although for some the changes in phosphopeptide abundance in response to DNA damage were not within the statistical threshold used here (Table S1). Examples included phosphorylation of the NHEJ component LIG4 (AT5G57160, S1058, S1168), KU70 (AT1G16970, S553) and RETINOBLASTOMA RELATED 1 (AT3G12280, S898).

### The response to DNA damage is dependent on ATM

**Figure 1:**
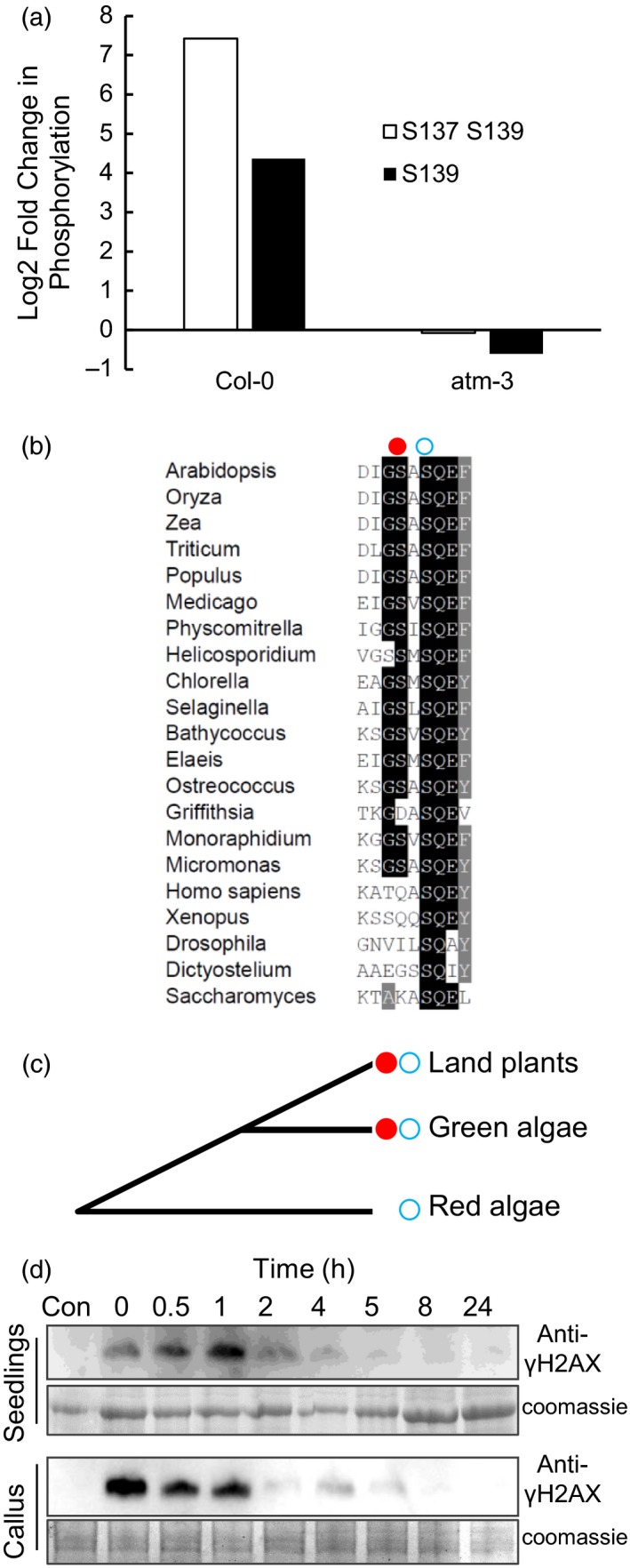
Tandem C‐terminal serine residues in plant H2AX are phosphorylated in response to genotoxic stress. (a) Fold‐induction of S137/S139 dual phosphorylation and S139 mono‐phosphorylation in Arabidopsis cell culture exposed to X‐rays (100 Gy). No significant induction was observed in atm‐3 mutants. (b) Protein sequence alignment of H2AX C‐termini from plant and algae species showing conservation of the dual S137/139 phosphorylation motif in green algae and land plants. Closed circles represent S137. Open circles represent S139. (c) Phylogeny indicating the presence of dual C‐terminal serine residues in the C‐terminus of H2AX in the plant lineage. Closed circles represent S137. Open circles represent S139. (d) SDS‐PAGE and western blot analysis of H2AX phosphorylation using phospho‐specific antisera raised to the dual phosphorylated H2AX C‐terminus (S137/S139) from Arabidopsis. The appearance of phosphorylated H2AX in 7‐day‐old Arabidopsis seedlings and cell culture was monitored at the time points indicated during recovery from a 100 Gy X‐ray dose.

The majority of changes observed in X‐rayed wild‐type lines were absent or severely attenuated in *atm* mutants, with 68% of phosphopeptides (153 peptides) that displayed significant changes in abundance upon irradiation of wild‐type showed no significant change in *atm* mutant lines (Table S2; FDR <0.05; Fold Change >1.5). For example, the X‐ray induced increase in histone H2AX phosphorylation was not observed in *atm‐3*, indicating a requirement for ATM for coordinating responses to DSBs in the timeframe of this study (Figure 1a). It remains possible that ATR may phosphorylate H2AX at later time points, as observed in the ATM‐independent H2AX phosphorylation in *mre11* and *rad50* mutants (Amiard *et al*., 2010). ATM and ATR phosphorylation occurs on SQ or TQ sites. Of the 254 phosphorylation sites identified in X‐ray responsive peptides, 4% were SQ/TQ, with SP/TP and SD/SE each accounting for ~30% of DNA damage‐induced changes in phosphorylation (Table S2). Sites of SE/D phosphorylation displayed a high incidence of acidic residues, and 72% of ATM‐dependent DNA damage responsive SE/D peptides contained an acidic residue at the +3 position after the phosphosite (Figure S4). SxxD/E forms the canonical site for casein kinase phosphorylation (Marin *et al*., 1994) and was also observed in the previous analysis of the Arabidopsis DNA damage response (Roitinger *et al*., 2015). The high incidence of ATM‐dependent phosphorylation on sites other than the canonical ATM/ATR targets is consistent with ATM activation of protein kinase‐mediated signalling cascades during the 100 min 100 Gy irradiation. Potential direct targets of ATM include the transcriptional co‐repressor LEUNIG (AT4G32551.2, LUG) which displayed an ATM‐dependent 44‐fold increase in S657Q658 phosphopeptide abundance in response to X‐rays (Table 1), in addition to the previously identified 2‐fold increase in T308 (Roitinger *et al*., 2015). LEUNIG has functions in vegetative plant development (Stahle *et al*., 2009), in addition to well characterized activities in the development of floral tissues, and the results presented here provides potential mechanisms for control of plant growth in response to genotoxic stresses. Meristem integrity is maintained in part by the hypersensitivity of stem cell initials to DNA damage and the low mitotic activity of the quiescent centre which protects these progenitor cells from the mutational effects of DNA damage (Fulcher and Sablowski, 2009; Heyman *et al*., 2014). Brassinosteroid signalling controls cell division in the quiescent centre (Heyman *et al*., 2013) and significantly, BIM2 (AT1G69010), a component of brassinosteroid signalling, displayed 58‐fold ATM‐dependent increase in S184Q185 phosphorylation in wild‐type lines upon irradiation, with no significant change observed in *atm* mutants (Table 1). Several unknown factors displayed ATM‐dependent increases in phosphopeptide abundance following X‐irradiation, including AT1G30240 that displayed a 10‐fold induction of T773Q774 phosphorylation (Tables 1 and S2). This protein displayed significant sequence homology to mammalian proline‐, glutamic acid‐, and leucine‐rich protein 1 (PELP1) that is a substrate for ATM, ATR and DNA‐dependent protein kinase and which is involved in p53‐mediated DNA damage signalling (Nair *et al*., 2014).

**Table 1:** Phosphopeptides displaying increased abundance in plant tissue exposed to X‐rays

AGI	Peptide sequence (S/T phosphosites)	Phosphosite (putative ATM target)		Gene	Fold change X‐ray/no X‐ray	
		Site	Site		WT	atm‐3
AT1G08880 AT1G54690	NKGDIG**S**A**S**QEF	S137	**S139**	HISTONE H2AXA or H2AXB	171.5	ns
AT1G69010	AITGISIE**S**QPELDDK	**S184**		BES1‐INTERACTING MYC‐LIKE 2 (BIM2)	58.41	ns
AT4G32551	FVEDGSLDDNVESFL**S**QEDGDQR	**S657**		LEUNIG (LUG)	43.81	ns
AT2G18410	DSDDEHPD**S**DEDPDDDLDI	S364		ELONGATOR COMPLEX PROTEIN 5 (ELP5)	37.29	ns
AT3G56150	FFTQVGSE**S**EDE**S**DYEVEVNEVQNDDVNNR	S13	S17	EUKARYOTIC TRANSLATION INITIATION FACTOR 3C (EIF3C)	24.63	ns
AT1G08880 AT1G54690	NKGDIGSA**S**QEF	**S139**		HISTONE H2AXA or H2AXB	20.61	ns
AT2G29190	NNLSPSASQGIGAPSPYSYAAVLGSSL**S**R	S265		PUMILIO 2 (PUM2)	14.41	ns
AT3G15040	**S**LAQSSLLSC**S**VLEGAGR	S204	S214	senescence regulator	14.35	ns
AT3G27530	LIELGVDVDKLLEDIGDESEAQAE**S**EED	S911		GOLGIN CANDIDATE 6 (GC6)	13.05	ns
AT2G45460	IVSVASNA**S**QDIK	**S21**		SMAD/FHA domain‐containing protein;	11.96	ns
AT1G17210	LGDSQDRVSQ**S**VVR	S769		IAP‐LIKE PROTEIN 1 (ILP1)	11.88	ns
AT1G30240	DGYEEVVSG**T**QEGEDLAVK	**T771**		proline‐, glutamic acid‐/leucine‐rich protein	10.12	ns
AT2G26530	LTVPFDWEETPG**T**PR	T60		Pheromone receptor‐like protein AR781	10.12	ns
AT1G67630	DVDMLLDGVQEDTEEIVTTP**T**NK	T115		DNA POLYMERASE ALPHA 2 (POLA2)	7.59	ns
AT1G76380	QE**S**DGEEPVSLSQQPK	S249		DNA‐binding bromodomain‐containing protein	7.31	ns
AT1G36730	NVTPFIEWLQNAE**S**E**S**EEE	S434	S436	Translation initiation factor IF2/IF5	6.71	ns
AT1G17210	FNAEQGIS**S**INDGEEVLNTETVTAQGR	S665		IAP‐LIKE PROTEIN 1 (ILP1)	6.22	2.45
AT1G36730	NVTPFIEWLQNAESE**S**EEE	S436		Translation initiation factor IF2/IF5	6.07	ns
AT4G35300	HEDWDEENLVGEGEDYPSDHGDD**S**EDDLH**S**PLISR	S361	S367	TONOPLAST MONOSACCHARIDE TRANSPORTER2 (TMT2)	5.98	10.2
AT3G46510	GRVDV**S**DDELYEDLQSLCNK	S147		PLANT U‐BOX 13 (PUB13)	5.94	ns
AT1G24300	ELASDNSIPL**S**PQWLYTK	S33		GYF domain‐containing protein	5.61	2.40
AT1G56340	DAPAESDAEEEAEDDDNEGDD**S**DNESKSEETK	S397		CALRETICULIN 1A (CRT1a)	5.55	ns
AT1G33680	EVNISG**S**QNEGEDDSKETNDVVAQK	**S154**		KH domain‐containing protein	5.55	ns
AT1G19350	I**S**NSAPVTPPVSSPT**S**R	S191	S205	BRI1‐EMS‐SUPPRESSOR 1 (BES1)	5.07	ns
AT4G27450	VD**S**EGVLCGANFKVDVYNR	S219		Aluminium induced protein	5.05	ns
AT2G18410	GGEIIYFRD**S**DDEHPDSDEDPDDDLDI	S357		ELONGATOR COMPLEX PROTEIN 5 (ELP5)	4.82	ns
AT4G18890	LPFFHGNSI**S**APV**T**PPLAR	S139	T153	BES1/BZR1 HOMOLOG 3 (BEH3)	4.58	ns
AT5G38840	KGIVEDEEDL**SS**DEDDFYDR	S372	S373	SMAD/FHA domain‐containing protein	4.57	ns

### Comparison of transcriptional and post‐transcriptional responses to high energy irradiation

The ATM‐dependent transcriptional response to DNA damage is well characterized in plants and results in upregulation of genes involved in genome maintenance, chromatin remodelling and DNA metabolism, while cell‐cycle factors are repressed (Culligan *et al*., 2006). Here, comparison with the published transcriptional responses of 6‐day old seedlings from 20 min up to 24 h after treatment with 100 Gy 100 min gamma‐rays (Bourbousse *et al*., 2018) revealed less than 8% overlap between the previously reported 2177 transcriptional targets and 180 post‐translational targets of the DNA damage response identified in Arabidopsis in the present study (Table S3). Factors identified here that were previously reported as responding transcriptionally include an uncharacterized protein that contains a Forkhead‐associated (FHA) domain (IPR000253, AT2G45460.3) and a structural maintenance of chromosomes (SMC) motif. AT2G45460.3 was previously identified as transcriptionally induced in response to gamma irradiation and hydroxyurea (Culligan *et al*., 2006; Yi *et al*., 2014) and here displayed ATM‐dependent S29Q30 phosphorylation (Table 1), in addition to previous reports of serine phosphorylation on residues 582 and 772 in response to genotoxic stress (Roitinger *et al*., 2015). The low level of overlap between transcriptionally responsive genes and proteins modified post‐translationally may reflect the differences in tissues and irradiation between studies, but is also consistent with regulation of the majority of plant DNA damage response factors at either at the level of transcription or post‐translationally, rather than both.

### Gene ontology of DNA damage responses

The post‐transcriptional DNA damage response identified here was further analyzed for enrichment in functional categories. Peptides with X‐ray dependent changes displayed significant enrichment in factors associated with post‐transcriptional regulation (GO:0010608) and epigenetic regulation (GO:0040029) (Table 2; FDR <0.05). Furthermore, the post‐translational response of callus cells to genotoxin stress included proteins associated with metal ion responses (GO:0010038) including cadmium (GO:0046686) (Table 2; FDR <0.05) (Sarry *et al*., 2006). For example, the phytochelatin synthase glutathione gamma‐glutamylcysteinyltransferase 1 (CAD1, AT5G44070), which is required for resistance to cadmium, displayed a four‐fold increase in phosphorylation of S352 upon genotoxin treatment. This change in phosphopeptide abundance was independent of ATM and may represent oxidative stress resulting from X‐irradiation. Significant enrichment was also observed for proteins associated with brassinosteroid mediated signalling (GO:0009742) including BES1/BZR1 HOMOLOG 3 (BEH3; AT4G18890) and BES1‐INTERACTING MYC‐LIKE 2 (BIM2, AT1G69010) (Tables 1 and 2).

**Table 2:** Gene ontology of post‐translational responses to X‐rays

Gene ontology	*P*	*n*	GO
Post‐transcriptional regulation of gene expression	6.58E‐04	13	GO:0010608
Regulation of macromolecule metabolic process	1.71E‐03	47	GO:0060255
Cellular response to organic cyclic compound	2.01E‐03	10	GO:0071407
Regulation of gene expression, epigenetic	3.27E‐03	11	GO:0040029
Negative regulation of macromolecule metabolic process	5.24E‐03	16	GO:0010605
Negative regulation of gene expression	5.40E‐03	14	GO:0010629
Regulation of metabolic process	7.79E‐03	47	GO:0019222
Negative regulation of metabolic process	1.21E‐02	16	GO:0009892
Regulation of primary metabolic process	1.53E‐02	44	GO:0080090
Response to chemical	1.92E‐02	41	GO:0042221
Brassinosteroid mediated signalling pathway	2.06E‐02	6	GO:0009742
Steroid hormone mediated signallling pathway	2.06E‐02	6	GO:0043401
Response to steroid hormone	2.06E‐02	6	GO:0048545
Cellular response to steroid hormone stimulus	2.06E‐02	6	GO:0071383
Cellular response to brassinosteroid stimulus	2.76E‐02	6	GO:0071367
Response to cadmium ion	2.83E‐02	12	GO:0046686
Regulation of translation	3.10E‐02	10	GO:0006417
Regulation of cellular amide metabolic process	3.22E‐02	10	GO:0034248
Response to metal ion	3.50E‐02	14	GO:0010038
Negative regulation of nitrogen compound metabolic process	4.78E‐02	12	GO:0051172

### Functional role of phosphorylation in the response to DNA damage

The functional significance of phosphorylation in the plant DNA damage response is largely uncharacterized, with post‐translational regulation of SOG1 by ATM providing the only example to date of the role of protein phosphorylation in plant resistance to genotoxic stress (Yoshiyama *et al*., 2013, 2017). C‐terminal phosphorylation of H2AX is a conserved response to genotoxic stress across eukaryotes, although the function of this phosphorylation event differs significantly between organisms and remains to be established in plants. Here, MS analysis identified extensive ATM‐dependent changes in H2AX phosphorylation in response to X‐rays, with no significant response observed in the *atm‐3* mutant lines (Table 1). Arabidopsis H2AX contains a well conserved SQEF C‐terminal motif that is phosphorylated in response to DNA damage, largely dependent on ATM (Friesner *et al*., 2005). Consistent with this, our MS analysis identified a 21‐fold ATM‐dependent increase in S139 in X‐ray‐treated wild‐type plants but not *atm‐3* mutants (Figure 1a). However, a significantly greater increase was seen in the dual phosphorylated S137 S139 C‐terminal motif, with a 171‐fold increased level of phosphopeptide in X‐ray‐treated wild‐type plants, and no significant induction in *atm‐3* mutants (Figure 1a). S137 phosphorylation occurs on a non‐canonical ‘SA’ motif, and is therefore unlikely to be a direct target of ATM. Dual S137 S139 phosphorylation was previously detected less than 20 min after exposure to gamma irradiation (Roitinger *et al*., 2015). However, phospho‐S137 was not detected in the absence of phospho‐S139 in either study, suggesting that phospho‐S139 H2AX forms a substrate for a second phosphorylation event by an unidentified kinase. While phosphorylation of the histone C‐terminal H2A isoform H2AX is a conserved response to induction of DSBs in eukaryotes, with S139 phosphorylation present in plants, mammals and yeast, the S137 site is conserved only in plant species. Database analysis identified that the dual serine motif was present in all plant species investigated including *Physcomitrella patens* and green algae, but not red algae or non‐plant species included in this analysis (Figure 1b). Thus, plants contain a unique conserved ‘GSXSQEΦ’ tandem phosphorylation site motif, suggestive that this feature has evolved in the plant lineage (Figure 1c). Polyclonal antiserum was raised to the tandem phosphorylated peptide, affinity purified with the phosphopeptide and depleted against the non‐phosphopeptide, and detected mono‐ and dephosphorylated peptides (Figure S3). SDS‐PAGE and western blot analysis notably showed a more rapid induction and turnover of H2AX phosphorylation in cultured cells relative to seedlings after X‐ray exposure (100 Gy in 100 min) (Figure 1d).

### Isolation of *h2ax* null mutant lines

**Figure 2:**
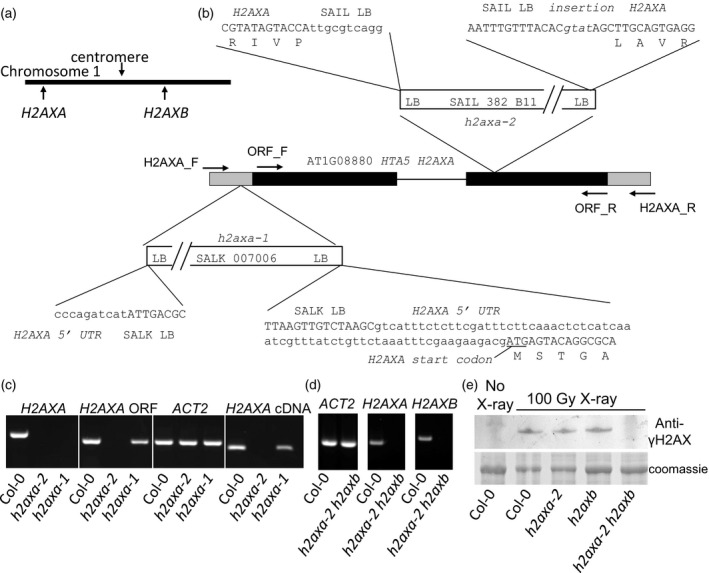
Isolation of null *h2axa* and *h2axa h2a2b* mutant lines. (a) Schematic showing the relative positions of *H2AXA* and *H2AXB* on Arabidopsis chromosome 1. (b) Schematic of the *H2AXA* gene showing the location and border sequences of T‐DNA insertions in the *h2axa‐1* and *h2axa‐2* mutant lines. Exons are shown as black boxes, introns as lines and untranslated regions are represented as grey boxes. (c) Isolation of the null *h2axa‐2* mutant line and PCR screening of wild‐type (Col‐0), *h2axa‐1* and *h2axa‐2* mutants. Lines were analyzed for the presence of the *H2AXA* full length gene (including UTRs), the *H2AXA* open reading frame (ORF), and RT‐PCR detected expression of *ACTIN2* (*ACT2*) and *H2AXA*. (d) Isolation of a null *h2axa h2a2b* double mutant line. PCR analysis of genomic DNA isolated from wild‐type and the *h2axa h2a2b* double mutant line confirmed that both *H2AX* genes were disrupted in the mutant line. (e) H2AX phosphorylation is abolished in the *h2axa‐2 h2axb* double mutant. SDS‐PAGE and western blot analysis of H2AX phosphorylation in Col‐0, *h2axa‐2*,* h2axb* and *h2axa‐2 h2axb* lines. Analysis of Arabidopsis 7‐day seedlings 3 h after exposure to 100 Gy X‐ray using phospho‐specific antisera raised to the dual phosphorylated H2AX C‐terminus (S137/S139) confirmed that no H2AX phosphorylation was detected in *h2axa h2a2b*. Lane 1‐unirradiated Col‐0 control. Representative of four independent biological replicates.

H2AX is a major target of ATM‐dependent phosphorylation, suggesting roles for γ‐H2AX in regulating the DNA damage response and maintaining plant genome stability under conditions of stress (Friesner *et al*., 2005). We therefore investigated the functional significance of H2AX phosphorylation on the dual S137 S139 motif. Arabidopsis possess two H2AX isoforms, termed A and B, located on chromosome 1 (Figure 2a). H2AX mutants and silenced lines have been previously reported as mildly hypersensitive to X‐ray irradiation and bleomycin and defective in DSB repair (Huefner *et al*., 2009; Lang *et al*., 2012; Qi *et al*., 2016), although the role of phosphorylation in H2AX function was not reported. Previous studies have suggested both possible functional redundancy and distinct functions for *H2AXA* and *H2AXB* (Huefner *et al*., 2009; Lang *et al*., 2012; Qi *et al*., 2016). The previously published *h2axa‐1* mutant line (SALK_007006, *hta5*) retained some H2AXA expression, although at a lower levels than wild‐type lines (Huefner *et al*., 2009). In the present study, a second allele, *h2axa‐2* (SAIL_382_B11) was isolated, containing a T‐DNA insertion near the beginning of the second exon (Figure 2b) which abolished *H2AXA* expression in homozygous lines (Figure 2c). The *h2axa‐2* allele was crossed into the previously reported *h2axb‐1* mutant line [SALK_012255, *hta3* (Huefner *et al*., 2009; Qi *et al*., 2016)] to generate double mutant plants. In the *h2axa‐2 h2axb* line no *H2AX* transcript was observed (Figure 2d), consistent with null mutations in both *H2AX* genes. The absence of detectable H2AX phosphorylation in the *h2axa‐2 h2axb* plants was confirmed using western blot analysis of X‐ray‐treated plants (Figure 2e).

### The roles of H2AX phosphorylation in plant responses to genotoxic stress

**Figure 3:**
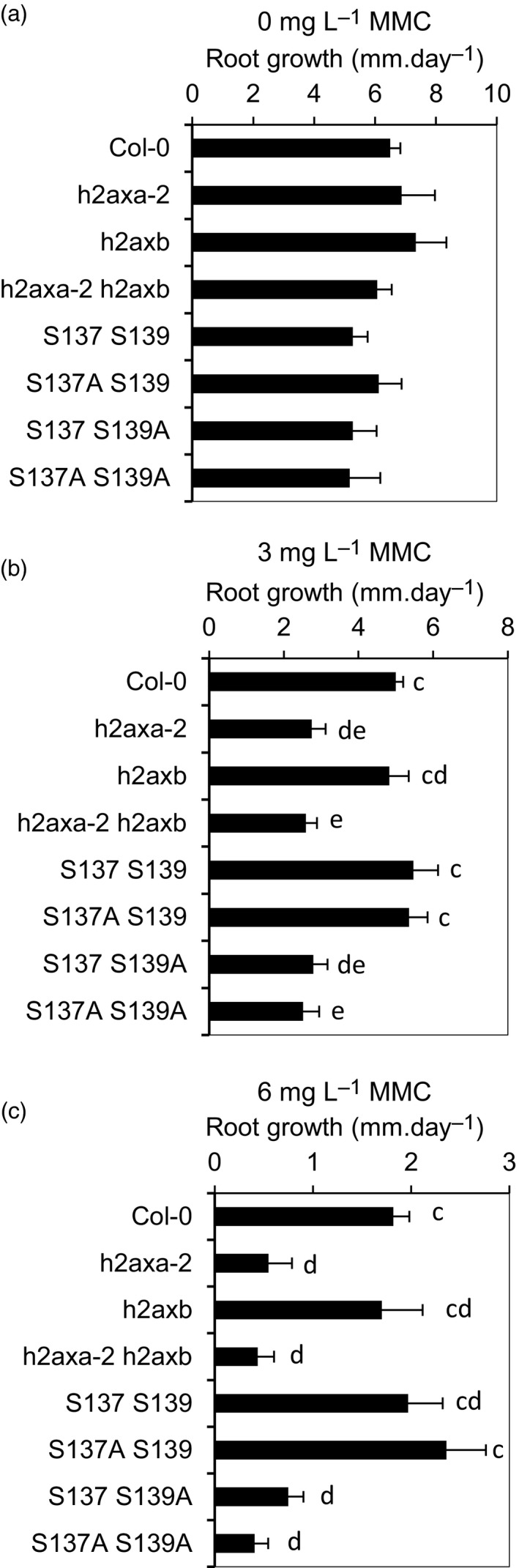
Phosphorylation of H2AX is required for resistance of plant growth to genotoxic stress. Root growth sensitivity of wild‐type, *h2axa‐2, h2axb, h2axa‐2 h2axb* mutant lines, and the *h2axa‐2 h2axb* double mutant line complemented with either wild‐type *H2AXA* or with phospho‐mutated *H2AX* constructs to the interstrand crosslinking reagent mitomycin C (MMC). Germinated 3‐day seedlings were plated on MS plates containing the stated concentration of MMC and grown vertically. *h2axa‐2* and *h2axa‐2 h2axb* lines show hypersensitivity to mitomycin C and wild‐type sensitivity is restored by complementation of *h2axa‐2 h2axb* with constructs containing S139 but not mutated S139A. (a) Root growth in the absence of mitomycin C. (b) Growth of plants on 3 mg L^−1^ MMC. (c) Growth of plants on 6 mg L^−1^ MMC. Data were analyzed using one‐way ANOVA with Tukey's HSD post hoc test, with letters indicating distributions that are not significantly different. Error bars show SEM of >15 roots. Representative of four independent biological replicates and transgene expression levels in the different lines is provided in Figure S8.

The *h2axa‐2 h2axb* double mutant line was viable and fertile, with no obvious growth defects. Initially the physiological significance of the tandem H2AX phosphorylation was investigated by phenotypic analysis of null *h2ax* double mutants to a range of genotoxic stresses including UV‐C (photoproducts and DNA breaks), X‐rays (DSBs), mitomycin C (interstrand crosslinks) and hydroxyurea (an inhibitor of DNA replication by reducing dNTP levels). Genotoxin sensitivity was determined by quantitative growth analysis of root length and significant hypersensitivity of the double mutant line was observed in response to methyl methanosulphate (MMS) treatment (*P* < 0.05), X‐rays (*P* < 0.05) and mitomycin C (*P* < 0.01) in line with previous reports of plants deficient in H2AX (Huefner *et al*., 2009; Lang *et al*., 2012) (Figure 3a–c; Figure S5). However, the growth sensitivity of the *h2axb* mutant line mutant to Mitomycin C (MMC) was not significantly different to wild‐type, while *h2axa‐2* mutants displayed a response similar to the double mutant. This suggests that H2AXA plays a principle role in root growth resistance to mitomycin C, with little redundancy of function between H2AXA and H2AXB. This result is surprising, given the similarity between the two proteins, but may result from differential expression of the two genes, whereby *H2AXA* accounts for the major fraction of H2AX protein in root cells sensitive to MMC. GUS‐reporter expression indicated largely overlapping patterns of the two H2XAX isoforms, although differences were observed in young root tips from 6d germinated seedlings (Figure S6). *H2AXA:GUS* expression was greater than *H2AXB:GUS* in the columnar cells, whereas both promoters displayed activity in distal regions of the root tip (Figure S6). Given the major role of H2AXA in resistance to MMC, H2AXA was used in complementation studies to investigate the functional significance of H2AX phosphorylation.

To define the functional requirements and roles of specific S137 and S139 phosphorylation events in plant responses to DNA damage and replication stress, *h2axa‐2 h2axb* mutant lines were complemented with either wild‐type *H2AXA* or H2AX constructs with one or both of the phosphorylated serine residues replaced by alanine (H2AXA‐S139A, H2AXA‐S137A and the dual phospho‐null H2AXA‐S137A S139A). Complemented lines were analyzed for the ability of the modified H2AX constructs to restore a wild‐type level of genotoxin sensitivity to the *h2axa‐2 h2axb* double mutant background. Wild‐type sensitivity to mitomycin C was restored by the addition of H2AXA constructs containing the S139 site, but not the S139A mutated form of H2AX (Figures 3a–c, S7 and S8). This demonstrated dependence of the plant response to genotoxic stress on phosphorylation of H2AX on S139 indicated that, although highly conserved in plants and phosphorylated in response to X‐rays, the S137 residue was not required for plant responses to genotoxic stress.

### H2AX is required during germination

**Figure 4:**
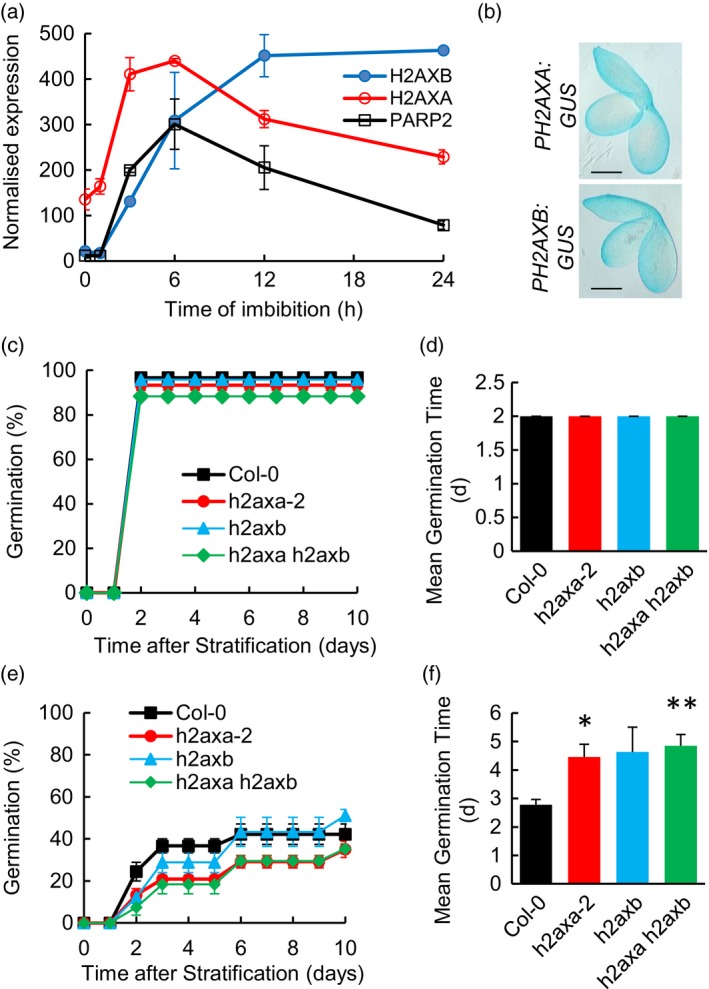
Germination of *h2axa‐2* and *h2axa‐2 h2axb* mutants is hypersensitive to seed ageing. (a) Expression of *H2AXA*,* H2AXB* and *PARP2* (AT4G02390, a marker of the transcriptional DNA damage response) during germination of unaged seed at 0–24 h imbibition (microarray data normalized using Affymetrix MAS5.0; Winter *et al*., 2007). (b) Expression of *H2AXA*:*GUS* and *H2AXB:GUS* in embryos at 16 h imbibition. (c–f) Germination of *h2axa‐2, h2axb, h2axa‐2 h2axb* and Col‐0 seeds was analyzed without (c, d) or with (e, f) accelerated ageing at 35°C and 83% relative humidity for 14 days. Seeds were stratified at 4°C for 48 h before being transferred to 22°C and then scored for radicle emergence on each day post‐imbibition. (c) Germination of control seeds without ageing treatment. (d) Control seeds (e) seeds after accelerated ageing for 14 days. (e) Germination of seeds after accelerated ageing. Error bars indicate the SEM of three replicates of 50 seeds each. *P < 0.05; **P < 0.01 (*T*‐test). (d, f) Mean germination time of *h2axa‐2, h2axb h2axa‐2 h2axb* and wild‐type seeds.

DNA double‐stranded break repair processes play particularly important roles at the seed stage of the plant lifecycle in response to naturally occurring DNA damage incurred as a consequence of desiccation−rehydration cycles and quiescence (Waterworth *et al*., 2015). ATM delays germination in the presence of DNA damage accumulated in aged seeds, facilitating repair processes that are required to promote germination and seedling establishment on soil (Waterworth *et al*., 2010, 2016). Analysis of microarray data (Winter *et al*., 2007; Bassel *et al*., 2008) indicated that both *H2AXA* and *H2AXB* were expressed throughout germination in Arabidopsis seeds, consistent with roles in germination (Figure 4a) and GUS‐reporter analysis demonstrated diffuse expression of *H2AXA* and *H2AXB* observed in cells throughout the embryo (Figure 4b). A role for H2AX in germination was further investigated using accelerated ageing of *h2axa‐2*,* h2axb* and *h2axa‐2 h2axb* mutant seed at 35°C to a relative seed moisture content of 10.8%. While no difference in germination was observed in the absence of seed ageing (Figure 4c,d), ageing resulted in delayed radicle emergence in the wild‐type seeds, but these effects were significantly more severe in *h2axa‐2 h2axb* mutants, which took on average 2 days longer to germinate (Figure 4e,f; *P* < 0.05). However, the sensitivity of the *h2axb* mutant line seed ageing was not significantly different from wild‐type, in contrast to that of *h2axa‐2* deficient seed (Figures 4e,f and S9). This identifies a physiological role for H2AX in maintenance of seed germination vigour which progressively increases in importance as seed quality deteriorates.

## Discussion

DNA damage responses play crucial and diverse roles in plants, promoting genome stability, cell‐cycle control and maintenance of meristem tissues (Waterworth *et al*., 2011). In eukaryotes the ATM and ATR protein kinases play central roles in orchestration of these processes through initiation of phosphorylation signalling cascades. Although extensive post‐translational DNA damage signalling networks have well characterized roles in mammals and yeast, their functional significance remains much less well defined in plants, with SOG1 phosphorylation being the major factor studied to date (Yoshiyama *et al*., 2017; Ogita *et al*., 2018). Moreover, the absence of several identifiable homologues of key mammalian DNA damage response factors in plants points to significant divergence of signalling pathways (Hu *et al*., 2016). Here, we employed a quantitative phosphoproteomic approach to elucidate ATM signalling and DNA damage responsive phosphoproteins in callus cells. MS analysis identified 219 previously unidentified phosphorylation targets and extensive post‐translational plant DNA damage responses to DSBs. The functional significance of phosphorylation in the plant DNA damage response was illustrated by complementation analysis of *h2ax* mutants, revealing the requirement of ATM‐dependent H2AX phosphorylation for plant growth under genotoxic stress.

Genome stability is important for plant growth and development and cells require rapid and effective DNA damage responses in order to prevent lesions causing mutations during DNA replication and mitosis that can be transmitted to the daughter cells and progeny plants (Ries *et al*., 2000). Here, phosphoproteomic analysis of an X‐irradiated callus population revealed many previously unknown candidate components of the plant DNA damage response, significantly enriched in proteins related to abiotic stress including metal ions and cadmium (Sarry *et al*., 2006). This may reflect that soil pollutants are a major cause of genotoxic stress in plants, and cadmium in particular was previously reported to induce DNA fragmentation and genome instability (Kovalchuk *et al*., 2001; Fojtova *et al*., 2002). Proteins with phosphopeptides that changed in abundance in X‐ray‐treated cells were enriched in stress response factors and included numerous transcription factors, although as noted previously (Roitinger *et al*., 2015), the SOG1 transcription factor is recalcitrant to MS analysis of trypsin digests and was not identified in this analysis. The extensive range of substrates identified here indicates that DNA damage responsive signalling involves a wide network of cellular factors. In addition to transcription factors, targets of genotoxin signalling include proteins with roles in translation, RNA stability and redox regulation.

ATAXIA TELANGIECTASIA MUTATED predominantly mediates the transcriptional DNA damage response, while both ATM and ATR activate cell death, cell‐cycle checkpoints and endoreduplication in flowering plants (Hu *et al*., 2016). Here, the majority (68%) of the identified post‐translational responses were dependent on ATM‐mediated signalling pathways, including potential direct targets of ATM. Previously, roles for ATM were identified in genotoxin‐induced changes in Histone H3 and H4 acetylation (Drury *et al*., 2012). A highly conserved response to DSBs is phosphorylation of histone H2AX at S139, resulting in recruitment of repair factors and formation of DNA repair foci (Natale *et al*., 2017). Interaction of MEDIATOR OF THE DNA DAMAGE CHECKPOINT 1 (MDC1) with phosphorylated H2AX potentiates the DNA damage response in mammals (Lukas *et al*., 2004), while downstream factors recruited to repair foci remain largely unknown in plants. However, fluorescence microscopy identified partial co‐localization of damaged‐induced γ‐H2AX foci in Arabidopsis with E2FA, a transcription factor involved in regulating genes required for DNA replication and cell cycle control (Lang *et al*., 2012), and recent studies localized RAD51, BRCA1 and RBR to γ‐H2AX foci (Biedermann *et al*., 2017; Horvath *et al*., 2017). Here we showed that H2AX phosphorylation during 100 Gy (100 min) genotoxin exposure was entirely dependent on ATM, with no significant phosphorylation observed in the *atm‐3* mutant. This contrasts with previous immunoblot and immunocytochemistry analyses of *atm‐2* mutants that showed H2AX phosphorylation post‐irradiation, although at reduced levels than wild‐type lines (Friesner *et al*., 2005). The *atm‐3* allele contains a T‐DNA insertion towards the 5′end of the gene, whereas the *atm‐2* allele is disrupted in the kinase domain near the 3′end, possibly retaining non‐kinase‐related functions of ATM. However, both alleles display similar low levels of fertility suggesting equivalent severity of mutation in the two *atm* alleles (Garcia *et al*., 2003; Waterworth *et al*., 2007). The C‐terminal domain of H2AX contains two serine residues (S137 and S139), which form a GSXSQEΦ consensus conserved in plant species and both of which are phosphorylated in Arabidopsis (Roitinger *et al*., 2015). X‐irradiation induced a 21‐fold increase in S139 phosphorylation, but notably a 171‐fold increase in C‐terminal phosphorylation of the dual serines in the SASQ motif, specific to plant H2AX. This is indicative of high levels of post‐translational modification in plant responses to genotoxic stresses and suggestive of functional roles for H2AX modification in plant growth under stress. H2AX RNAi lines were previously reported to be hypersensitive to genotoxins including the radiomimetic bleomycin and the topoisomerase inhibitor camptothecin (Lang *et al*., 2012). Analysis of the *h2axa‐1* and *h2axb* T‐DNA insertion lines revealed little hypersensitivity of root growth to gamma irradiation, although the *h2axa‐1* displayed residual expression (Huefner *et al*., 2009). However, both mutant lines, and the double *h2axa‐1 h2axb* mutant displayed altered repair at a zinc finger nuclease induced break, indicative of altered activity of both NHEJ and HR pathways (Qi *et al*., 2016). In the present study, complementation of the null *h2axa‐2 h2axb* mutant line with phosphorylation site‐mutant versions of H2AXA identified a specific requirement of phosphorylation for H2AX function. Given the conservation of tandem C‐terminal serine residues, the function of each residue was investigated in complementation studies with plants lacking H2AX. Wild‐type levels of mitomycin C sensitivity were restored by constructs containing the S139 phosphorylation site, indicating that modification of this residue was necessary for H2AX function in the DNA damage response, and that S137 was not required for wild‐type levels of growth in the presence of the genotoxin. The particularly high sensitivity of *h2ax* mutant plants to the DNA crosslinking reagent MMC relative to other forms of DNA damage mirrors phenotypic analysis of mutants in homologous recombination (HR), including the main recombinase RAD51 and its paralogues (Bleuyard *et al*., 2005). Potential function for γ‐H2AX in promotion of HR activity in plants is supported by the recent finding that RAD51 colocalizes with γ‐H2AX in response to DNA damage (Biedermann *et al*., 2017). Taken together with the altered pattern in end‐joining products previously identified in H2AX‐deficient lines (Qi *et al*., 2016) and the differential sensitivity of *h2ax‐2* mutant seeds to ageing observed in the present study, these results indicate that in plants H2AXA influences multiple pathways for the repair of DSBs and is not redundant in function to H2AXB. These functions are likely to be linked to protein complexes nucleating at γ‐H2AX foci and future studies are required to determine the mechanisms of repair foci formation and their role in plant genome maintenance.

To further elucidate the physiological function of H2AX in plants, the sensitivity of mutants deficient in H2AX to seed ageing was investigated. Seed germination potential is important both to plant survival in the natural environment and optimal crop yields in agriculture, in particular under stress (Rajjou *et al*., 2012; Finch‐Savage and Bassel, 2016). Desiccation tolerant (orthodox) seeds, typical of plant species from temperate latitudes, accumulate high levels of DNA damage at this stage of their lifecycle (Waterworth *et al*., 2010, 2015, 2016). Here, mutant hypersensitivity to seed ageing identified a role for H2AX in germination and seed longevity, providing insight into the physiological significance of H2AX in responses to naturally occurring DNA damage. These results are consistent with important roles for ATM post‐translational signalling networks operating in seeds, supported by the known functions of ATM in germination and maintenance of genome integrity in seeds (Waterworth *et al*., 2016). ATM functions to modulate cell‐cycle activity in response to DNA damage, imposing a delay to germination, whereas H2AX is a repair factor and, like several other DNA repair genes, displays hypersensitivity to seed ageing (Waterworth *et al*., 2010, 2016; Córdoba‐Cañero *et al*., 2014). Phosphoproteome analysis of germinating *Lotus japonicas* also identified numerous phosphorylated ATM/ATR SQ consensus motifs (Ino *et al*., 2014), further supporting the importance of these post‐translational signalling networks at the seed stage of the plant lifecycle.

In conclusion, DSB DNA damage post‐translational signalling networks in plants are extensive and largely mediated by the sensor kinase ATM and downstream signalling pathways. Their functional significance is established by analysis of plants expressing phosphorylation site‐mutated H2AX proteins, a major ATM target, which display hypersensitivity to DNA damage. Future studies are required to further determine the roles of post‐translational regulation in plant responses to DNA damage, and the network of protein modifications identified here will facilitate a deeper understanding of how plants are able to maintain genome stability and promote growth under increased levels of environmental stresses. These responses are important for improvement of crop stress resistance under rapidly changing climates.

## Experimental procedures

### Arabidopsis growth and treatments

Arabidopsis Col‐0 and mutants were obtained from NASC or have been described previously: *atm‐3* (Waterworth *et al*., 2007), *h2axa‐1* (SALK_007006, *hta5*) and *h2axb‐1* mutant line (SALK_012255, *hta3*) (Huefner *et al*., 2009; Qi *et al*., 2016). A second allele, *h2axa‐2* (SAIL_382_B11) was obtained from NASC. Plants were grown on half‐strength Murashige and Skoog Basal Medium (½MS), 1% sucrose, 0.5 g L^−1^ MES and 0.8% plant agar (Duchefa) pH 5.7 under 16 h:8 h light dark cycles at 22°C. Root growth was measured by growing plants vertically in the surface of agar plates. Data was analyzed in SPSS using Levene's test for equality of variances and one way ANOVA with Tukey's HSD post hoc test. Cell callus was induced by transfer of Col‐0 or *atm‐3* leaf explants to Gamborg's B5 Medium including vitamins (Sigma G5893), 2% glucose, 0.5 g L^−1^ MES, 0.5 mg L^−1^ 2,4‐D, 50 μg L^−1^ kinetin, 0.8% plant agar (Duchefa) pH 5.7, 16 h:8 h light dark cycles at 22°C, replated every 2 weeks and used for proteomics analysis 5 weeks after initial transfer onto callus induction medium. qRT‐PCR analysis of representative callus tissue derived from Col‐0 and *atm‐3* leaf explants identified elevated expression levels of cell‐cycle regulators *CYCB1;1*,* CDKB2;1*,* CDKB2;1* and *CDKA;1* relative to 2 week seedlings (Figure S1).

### Nucleic acid purification, amplification and cloning

DNA procedures and bacterial manipulations used established protocols (Sambrook *et al*., 1989) and primer sequences are provided (Figure S2). RNA was isolated from plant tissues of flowering Arabidopsis using the SV total RNA isolation kit (Promega, Madison, WI, USA) according to the manufacturer's instructions and quantified by spectrometry (Thermo Fisher Scientific, Altrincham, UK). RT‐PCR was performed using Superscript II reverse transcriptase (Thermo Fisher Scientific, Altrincham, UK) for cDNA synthesis followed by amplification using iPROOF (Bio‐Rad, Watford, UK). PCR products were cloned using a TOPO‐TA cloning kit and *E. coli* TOP10 cells (Thermo Fisher Scientific, Altrincham, UK), and plasmid DNA was prepared using spin columns (Qiagen, Manchester, UK) prior to DNA sequencing (GATC Biotech, Ebersberg, Germany). Complementation of *h2ax* mutants used a *H2AXA* genomic clone in pCB1300 under the control of a 35S promoter and used for Arabidopsis transformation (Clough and Bent, 1998). pCB1300 was first modified to have a nos terminator inserted into the *Sal*I *Hin*dIII sites, amplified from pCB1381z with primers nosT f and nosT r. P35S was inserted to *Eco*RI *Bst*EII sites, amplified from pCB2300 with primers pCB2300 35S_LIC_f and pCB2300 35S_LIC_r. The *Ecl*136II site in pCB2300 35S_LIC_r was used for ligation‐independent cloning as described (De Rybel *et al*., 2011). For GUS expression pCB1381z was modified by ligation of pCB1381‐LIC‐*Eco*RI and pCB1381 LIC *Nco*I into after a double digest with *Eco*RI and *Nco*I and the *Ecl*136II site used for ligation‐independent cloning. H2AXA and H2AXB promoter regions were identified as the upstream 0.9 and 1 kb respectively, running up to the preceding gene. Transformed plants were selected on MS medium supplemented with hygromycin (40 mg L^*−*1^) and claforan (50 mg L^*−*1^). DNA extraction for PCR genotyping was performed by grinding plant tissue in shorty buffer (0.2 m Tris pH 9.0, 1% SDS, 0.4 m LiCl, 25 mm EDTA) in a 1.5 ml microfuge tube using a plastic micropestle. Cell debris was pelleted at 13 000 ***g*** for 5 min and the supernatant mixed 1:1 with 100% isopropanol and DNA recovered by centrifugation and dissolved in TE buffer.

### Protein preparation and in solution digestion

Plant tissue grown in culture was irradiated with 100 Gy using a 320 kV X‐ray irradiation system (NDT Equipment Services, Wellingborough, UK) at a dose rate of 1 Gy min^−1^ and immediately frozen in liquid nitrogen. Frozen tissue was ground to a fine power and proteins were solubilized in 10 m urea, 50 mm Tris pH 8. Insoluble material was removed by centrifugation at 10 000 ***g*** for 10 min. The supernatant was diluted to 2 m urea with dH_2_O and extracted with three volumes of methanol, one volume of chloroform and four volumes of dH_2_O. The sample was centrifuged for 1 min at 10 000 ***g*** and the aqueous phase removed. Protein was precipitated by addition of four volumes of methanol and incubation at −20°C for 1 h. The precipitate was recovered by centrifugation at 10 000 ***g*** for 10 min and allowed to dry at room temperature. Protein was solubilized in 1% sodium deoxycholate, 20 mm Tris‐HCl pH 8.0 at 90°C for 10 min. Samples were reduced with 10 mm dithiothreitol (DTT; 1 h, 56°C) followed by alkylation with 15 mm iodoacetamide (IAM; 45 min, RT). DTT was then added at 5 mm (45 min, RT) to quench any remaining IAM. Trypsin was added and samples left to digest overnight at 37°C.

### Sample desalting and phosphopeptide enrichment

Digested samples were desalted using Oasis HLB sample extraction columns (Waters, Manchester, UK) followed by their elution in phosphopeptide binding solution (65% (v/v) acetonitrile (ACN), 2% (v/v) trifluoroacetic acid (TFA), saturated with glutamic acid, pH 2–3). Samples were incubated with titanium dioxide beads (Glygen, Columbia, MD, USA) for 60 min at RT. The beads were then washed with binding solution, followed by wash solution 1 (65% (v/v) ACN, 0.5% (v/v) TFA), followed by wash solution 2 (65% (v/v) ACN, 0.1% (v/v) TFA). Enriched phosphopeptides were eluted from the beads by incubating with elution solution (300 mm ammonium hydroxide, 5% (v/v) ACN) for 5–10 min at RT (Robertson *et al*., 2015). Eluted phosphopeptides were then acidified to pH 2–3 using TFA, and desalted again using an in‐house 96‐well plate format for solid phase extraction (SPE).

### Phosphopeptide desalting using in‐house 96‐well format SPE

Desalting plates were generated by the addition of 1 mg OLIGO R3 beads (Life Technologies, Paisley, UK) to each well of a Corning 96‐well plate containing a 0.2 μm polyvinylidene difluoride (PVDF) membrane (Fisher Scientific, Loughborough, UK). Beads were firstly wet by the addition of 50% (v/v) ACN, which was removed by centrifugation (1 min, 360 *g*). Beads were equilibrated by the addition of 0.1% (v/v) formic acid (FA), the sample applied and then washed twice with 0.1% (v/v) FA. Following each step the liquid was removed by centrifugation (1 min, 360 ***g***). Samples were eluted in 50% (v/v) ACN, 0.1% (v/v) FA, collected via centrifugation (1 min, 360 *g*) and dried down to completion by vacuum centrifugation. For LC‐MS/MS analyses samples were resuspended in 0.1% (v/v) FA.

### Mass spectrometry

Digested samples were analyzed by LC‐MS/MS using an UltiMate^®^ 3000 Rapid Separation LC (RSLC, Dionex Corporation, Sunnyvale, CA, USA) coupled to an Orbitrap Elite (Thermo Fisher Scientific, Waltham, MA, USA) mass spectrometer. Peptide mixtures were separated using a gradient from 92% A (0.1% FA in water) and 8% B (0.1% FA in acetonitrile) to 33% B, in 44 min at 300 nl min^−1^, using a 250 mm × 75 μm i.d. 1.7 μm BEH C18, analytical column (Waters). Peptides were selected for fragmentation automatically by data dependant analysis, with multistage activation enabled for fragmentation of product ions resulting from the neutral loss of phosphoric acid (Schroeder *et al*., 2004).

### Data analysis

Tandem mass spectra were extracted using the *extract_msn* script (Thermo Fisher Scientific, Altrincham, UK) executed in Mascot Daemon (version 2.2.2.; Matrix Science, London, UK). Peak lists were searched against the UniProt *Arabidopsis thaliana* database (version ARATH, 2013‐05). Trypsin was included as the cleavage enzyme and a maximum of one missed cleavage was allowed. A peptide mass tolerance of 5 ppm and a fragment tolerance of 0.5 Da was included. Carbamidomethyl of cysteine was included as a fixed modification, with oxidation of methionine and phosphorylation of serine, threonine and tyrosine residues included as variable modifications. Data were validated using Scaffold (version 4.0.5, Proteome Software, Portland, OR, USA). Phosphopeptide identifications were accepted if established with at least 95% probability at the peptide level and at least 50% probability at the protein level and a threshold peptide ID score of 20 was applied.

### Label‐free quantification

The acquired data was analyzed using Progenesis (v4.1, Nonlinear Dynamics). The retention times for each sample were automatically aligned to a reference sample that was automatically selected using the software's algorithms. Protein quantitation was based on peak modelling and alignment to the reference sample. An aggregate data from all aligned runs was mapped to individual samples and used for ion abundance quantification and data normalized between runs (http://www.nonlinear.com/progenesis/). Features were automatically selected using all software defaults, and any features with ≥5+ charge state or less than three isotopic peaks were masked from further analysis. The software was used to generate a peak list that was searched against UniProt *Arabidopsis thaliana* (version ARATH, 2013‐05) using Mascot (v2.4, Matrix Science). The search parameters included a precursor tolerance of 5 ppm; a fragment tolerance of 0.5 Da; enzyme specificity for trypsin; one missed cleavage allowed; Carbamidomethyl of cysteine as a fixed modification, oxidation of methionine and phosphorylation of serine, threonine and tyrosine residues as variable modifications. Phosphopeptides with the highest protein match score were selected for further analysis where multiple ion charge fragments were identified. Data was analyzed using limma in R to determine peptides that displayed significant changes in abundance upon X‐irradiation using a linear model and an empirical Bayes method as described previously (Ritchie *et al*., 2015). Gene lists were analyzed using Araport11 v1.10.4 (araport.org) for enrichment in gene ontology terms with Holm‐Bonferroni correction for multiple testing.

### Antibody production

Phospho‐specific peptide antiserum was prepared to regions 132–142 (GDIGSpASpQEF) of Arabidopsis histone H2AX. Phosphopeptide synthesis, antisera generation and affinity purification were performed by the University of Dundee Antibody Production Unit (Dundee, UK). Antisera were purified using the phosphopeptide antigen and depleted of non‐phospho‐specific binding activity using the unmodified peptide. All antibody incubations were performed following a pre‐incubation with 10 μg of non‐phosphorylated peptide per microliter of purified antisera. Specificity was determined by ELISA (Figure S3), with purified antisera tested against non‐phosphorylated peptide, each of the mono‐phosphorylated forms and the di‐phosphorylated peptide and commercial anti‐phosphoS139 (ab11174, Abcam) used as a control. Peptides (200 μl at 2 mg ml^−1^ in PBS) were coupled to 1 mg bovine serum albumin (BSA) by addition of 5 mg 1‐ethyl‐3‐(‐3‐dimethylaminopropyl)carbodiimide hydrochloride (EDC) at room temperature for 2 h. A dilution series of 200 ng–2 pg coupled peptides was tested by ELISA using a 1:200 dilution of antisera, 1:10 000 anti‐sheep (or rabbit) HRP and colour development with 3,3′,5,5′‐tetramethylbenzidine (TMB).

### SDS‐PAGE and western blotting

Proteins were extracted from Arabidopsis tissue as described previously (Saleh *et al*., 2008). Protein concentrations were determined by the Bio‐Rad protein assay (Bio‐Rad Laboratories, Hemel Hempstead, UK) using BSA as a standard. Protein samples were separated by SDS‐PAGE (10% gel) and transferred to PVDF membrane (Bio‐Rad) for 1 h at 40 V. The blots were probed with anti‐Arabidopsis H2AX. The immune complexes were detected by alkaline‐phosphatase conjugated anti‐sheep IgG (Sigma‐Aldrich, Poole, UK) and developed using pre‐mixed BCIP/NBT solution (Sigma). Primary and secondary antisera were used at 1/1000 and 1/30 000 dilutions respectively.

### Seed germination and seed ageing

Seeds from all lines were grown and harvested simultaneously and stored at 15°C and 15% humidity for 2 months to allow after‐ripening. Germination tests and accelerated ageing were performed as described previously at a temperature of 35°C with a relative moisture content of 10.8% (Hay *et al*., 2003; Waterworth *et al*., 2016). Mean germination time was calculated as follows: the number of seeds germinated in the time interval since the previous observation is multiplied by the time point of that observation. The sum of these values for all observations is then divided by the total number of seeds germinated at the end point to give the mean time of germination.

## Data Statement

The mass spectrometry proteomics data have been deposited to the ProteomeXchange Consortium via the PRIDE (Vizcaino *et al*., 2016) partner repository with the dataset identifier PXD007100 and 10.6019/PXD00710 and is MIAPI compliant. All additional material and data reported in this manuscript is available from the corresponding author on request.

## Author Contributions

CEW, WMW and TN conceived the original research plans; CEW and SW supervised the experiments; WMW performed most of the experiments; JS performed the phosphoproteomic mass spectroscopy and MW and DW analyzed the proteomics data; CEW and WMW designed the experiments and analyzed the data; CEW and WMW conceived the project and wrote the article with contributions of all the authors.

## Conflict of Interest

The authors have no conflict of interest to declare.

## Supporting information


**Figure S1**. Callus tissue displays elevated levels of cell‐cycle associated transcripts.

**Figure S2**. Primers.

**Figure S3**. Specificity of anti‐H2AX phosphopeptide antisera.

**Figure S4**. Analysis of phosphosites.

**Figure S5**. Sensitivity of the *h2axa‐h2axab* mutants to genotoxins.

**Figure S6**. Analysis of H2AXA and H2AXB GUS‐transcriptional reporter lines.

**Figure S7**. Complementation of *h2axa h2axb* mutants with phosphorylation‐site‐mutated H2AXA.

**Figure S8**. *H2AXA* expression analysis of complemented *h2ax* mutants.

**Figure S9**. Germination performance of h2axa‐2, h2axb, h2axa‐2 h2axb and wild‐type lines.

**Table S1**. Phosphopeptides identified by mass spectroscopy.

**Table S2**. Phosphopeptides that display significant changes in abundance upon X‐ray treatment.

**Table S3**. Factors that display both transcriptional responses in gamma‐irradiated seedlings and post‐transcriptional responses in X‐ray‐treated callus tissue.
