# Supplementary figures and images for: Phosphoproteomic analysis reveals plant DNA damage signalling pathways with a functional role for histone H2AX phosphorylation in plant growth under genotoxic stress
Source: Plant J. 2019 Sep 10;100(5):1007–21. doi: 10.1111/tpj.14495 (PMC6900162; doi:10.1111/tpj.14495)

a

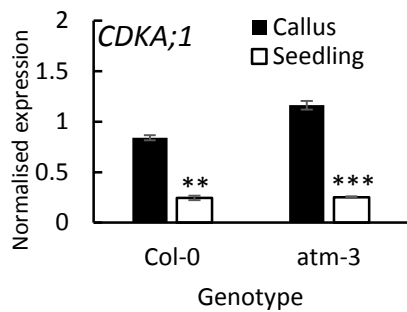

b

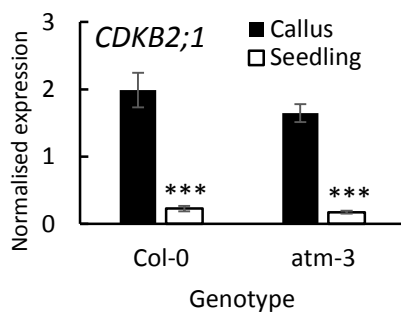

c

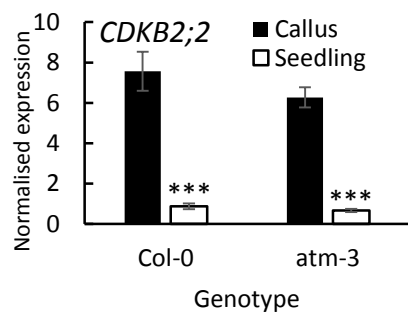

d

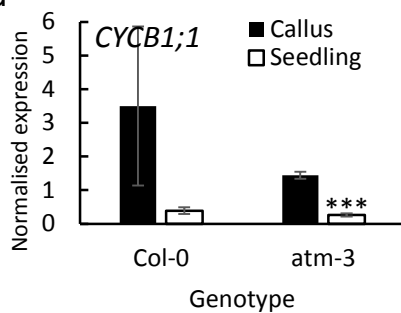

Supplement: Supplementary file 1 — Figure S1. Callus tissue displays elevated levels of cell‐cycle associated transcripts. [file TPJ-100-1007-s001.pdf]

a

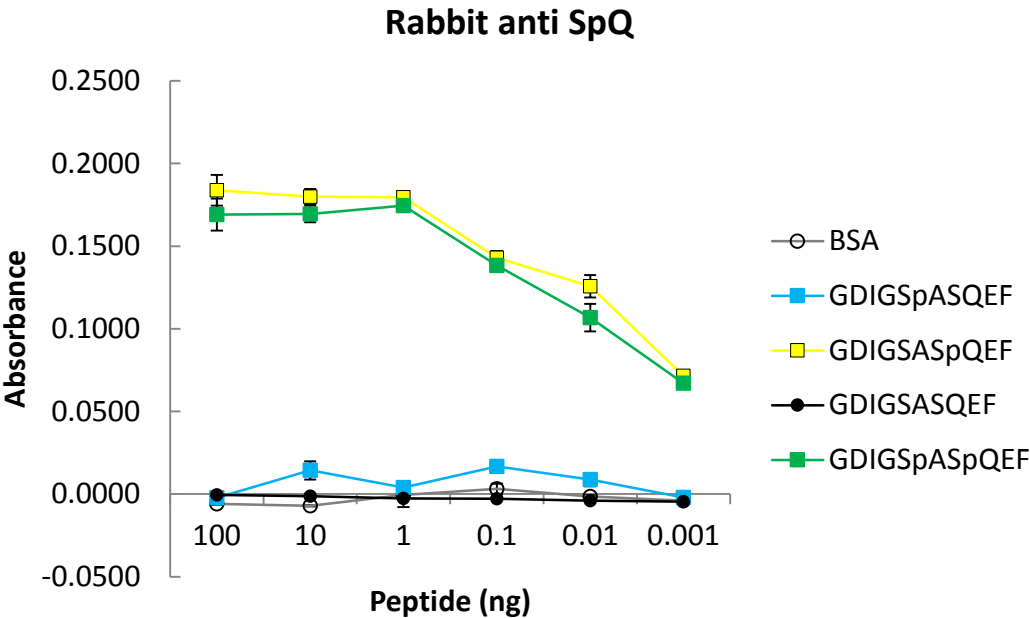

b

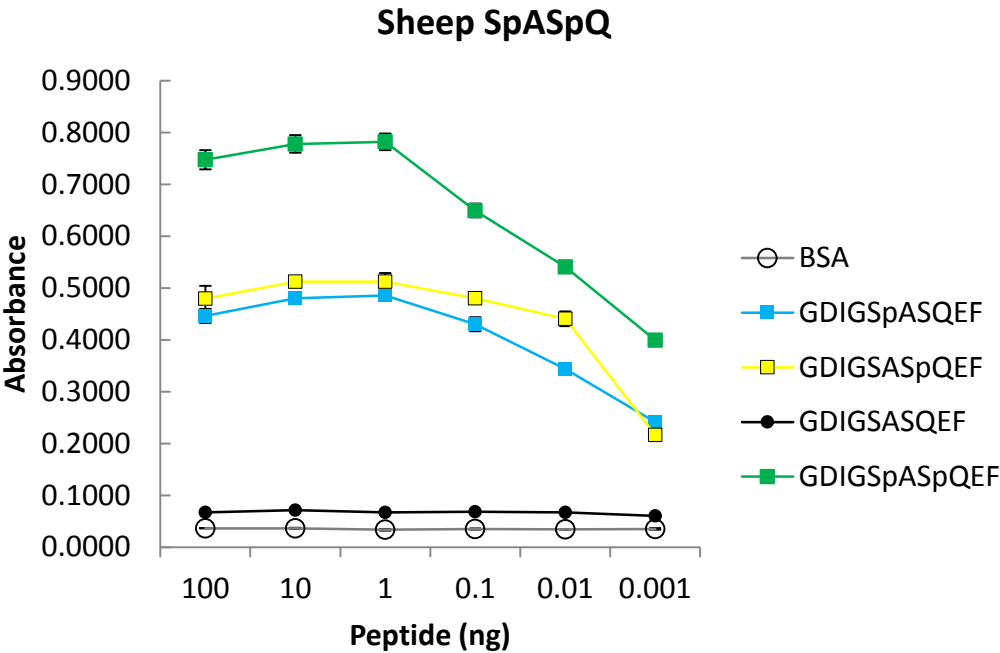

Supplement: Supplementary file 3 — Figure S3. Specificity of anti‐H2AX phosphopeptide antisera. [file TPJ-100-1007-s003.pdf]

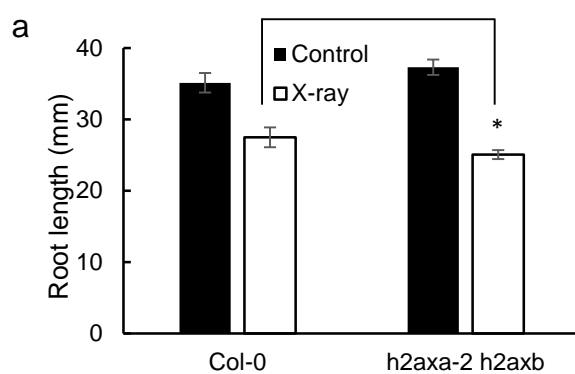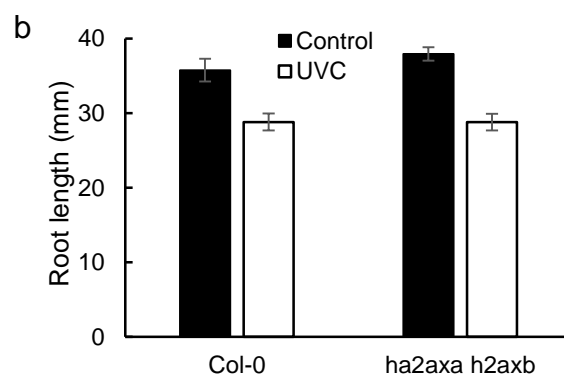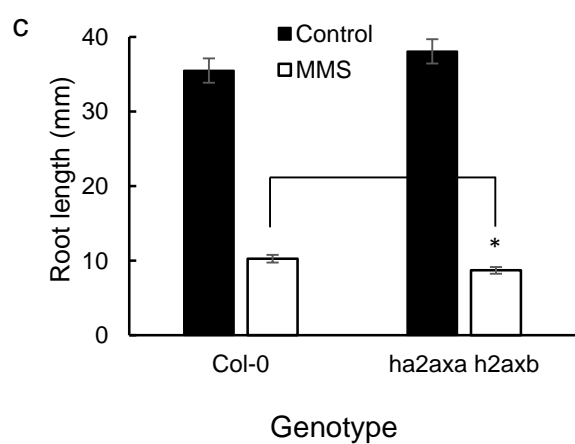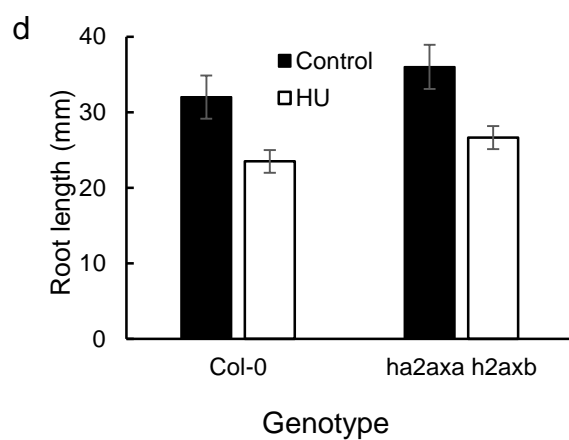

Supplement: Supplementary file 5 — Figure S5. Sensitivity of the h2axa‐h2axab mutants to genotoxins. [file TPJ-100-1007-s005.pdf]

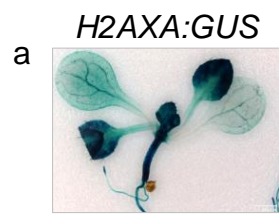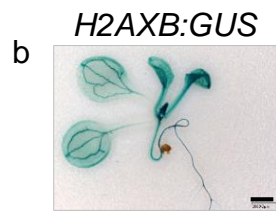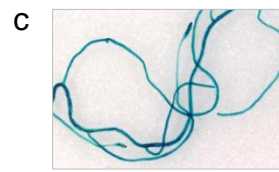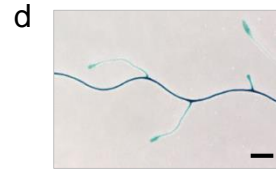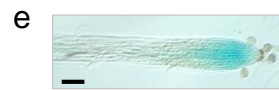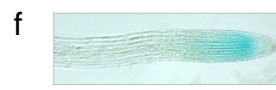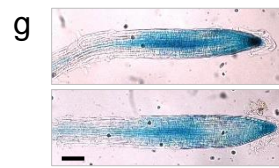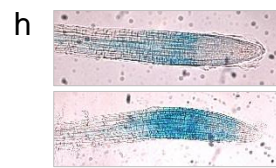

Supplement: Supplementary file 6 — Figure S6. Analysis of H2AXA and H2AXB GUS‐transcriptional reporter lines. [file TPJ-100-1007-s006.pdf]

a

0 mg.l<sup>-1</sup> MMC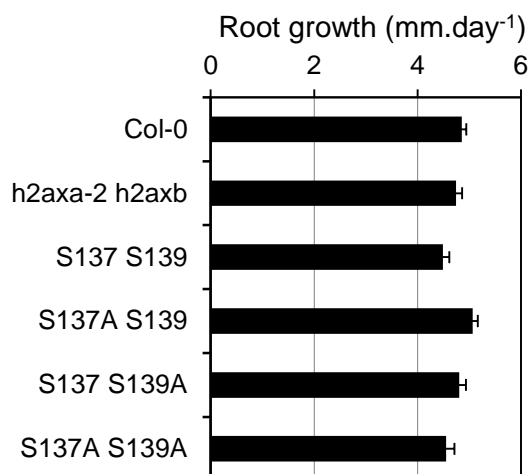

b

3 mg.l<sup>-1</sup> MMC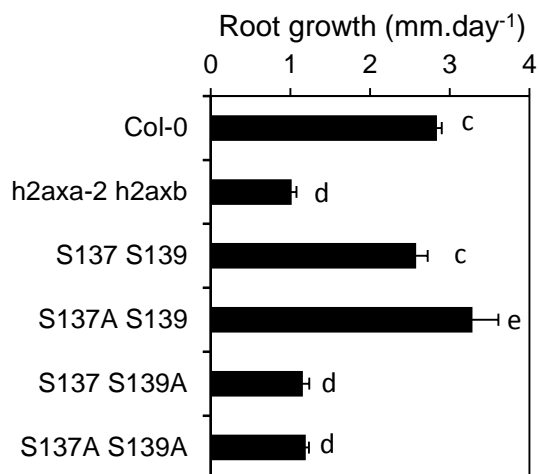

c

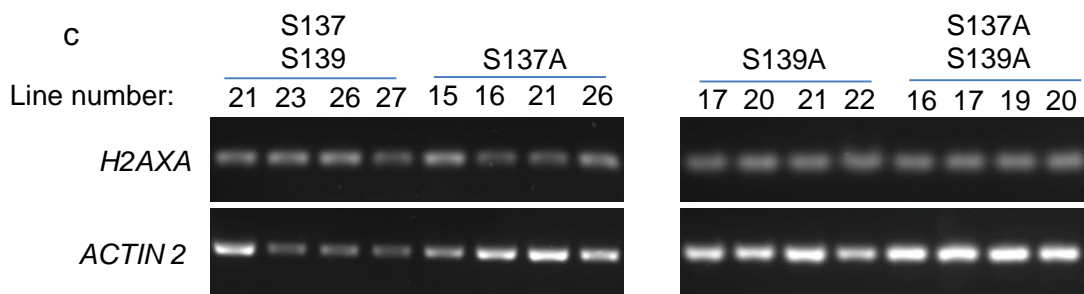

Supplement: Supplementary file 7 — Figure S7. Complementation of h2axa h2axb mutants with phosphorylation‐site‐mutated H2AXA. [file TPJ-100-1007-s007.pdf]

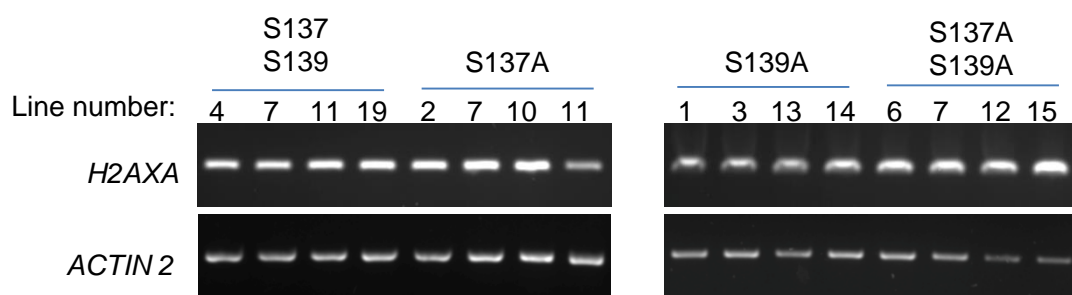

Supplement: Supplementary file 8 — Figure S8. H2AXA expression analysis of complemented h2ax mutants. [file TPJ-100-1007-s008.pdf]

a

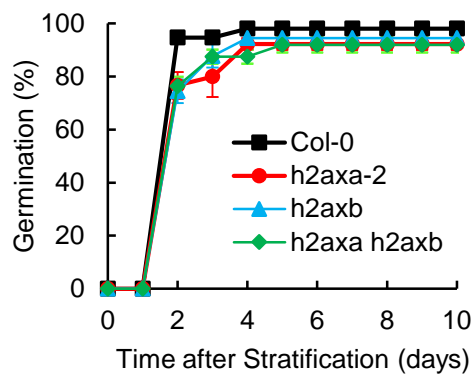

b

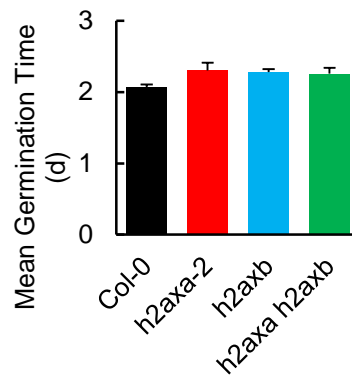

1 week ageing

Supplement: Supplementary file 9 — Figure S9. Germination performance of h2axa‐2, h2axb, h2axa‐2 h2axb and wild‐type lines. [file TPJ-100-1007-s009.pdf]
