# Supplementary material for: Phosphoproteomic analysis reveals plant DNA damage signalling pathways with a functional role for histone H2AX phosphorylation in plant growth under genotoxic stress
Source: Plant J. 2019 Sep 10;100(5):1007–21. doi: 10.1111/tpj.14495 (PMC6900162; doi:10.1111/tpj.14495)
Supplement: Supplementary file 2 — Figure S2. Primers. [file TPJ-100-1007-s002.pdf]

```
qCDKB2;1 F  TGGCTGCATATTTGCTGAAC
qCDKB2;1 r  AGGTTTGGAACAGCAGAGGA
qCDKB2;2 F  ATCTTTGCGGGAGACTCTGA
qCDKB2;2 r  GTTTTGCTGGCTCGTACTCC
qCDKaF,     GATACATGGCGTGGGGTAAC
qCDKaR,     GGCTCTTGCGTTGATTCTTT
qCYCB1_1 F, TGGTGCACTATTTGGCTGAG
qCYCB1_1 R, TCTTGTTGCTTCCATTGCTG

SALK_012255.55.25.x     h2axB   At1g54690
h2axB WT LP TCACTTTTTAGTTGGCCCATG
h2axB M RP  ATTGTGGAACAGAGAGCCATG

SALK_007006   H2AXA-1 hta5 At1g08880
SALK_007006LP ACATTGGGATGATCAATCCAC
SALK_007006RP TGTGTTTCAAAATGTTTCTGGG

SAIL_382_B11  H2AXA-2 At1g08880
SAIL_382_B11LP TTAGCGAGCTAATTCAGGTGC
SAIL_382_B11RP CCCTAAAGCCCACTCATCTTC

S137A gaaggagaagccgagTGgtcacctcagaactcctgagaagcagctccaatatctcctttgttcttg
S139A gaaggagaagccgagTGgtcacctcagaactcctgagcagcagatccaatatctcctttgttcttg
S137A S139A gaaggagaagccgagTGgtcacctcagaactcctgagcagcagctccaatatctcctttgttcttg
WT gaaggagaagccgagTGgtcacctcagaactcctgagaagcagatc
H2AfLIC  gacgacgacagagtaaaATGAGTACAGGCGCAGGAAG

H2AXA 0.9Kb      GACGACGACAGAGTCCAATCTGAGGCATGTTAGTTG
H2AX A GUS rev GAAGGAGAAGCCGAGTCGTCTTCTTCGAAATTTAGAACAG
H2AXB 1Kb      GACGACGACAGAGTGCCATCATCTTCTACGGTCATACC
H2AX B GUS rev GAAGGAGAAGCCGAGTTTTTGTTTATCGGGAAATTTGAAG

pCB1381 LIC EcoRi aattc tgacgacgacagagctcggcttctccttca
pCB1381 LIC NcoI gtaccacttcctcttcggctcgagacagcagcagtc
pCB2300 35S_LIC_r CAggtcacctgaaggagaagccgagctctgtcgtcgtcacccgggatctgcgaaagctc
pCB2300 35s For GATCGAATTCagagcagcttgccaacatgg
nosT F  gatgtcgacaggtgaccagctcgaatttc
nosT R  gataagcttcccgatctagtaacatagatg
```

Figure S2 Primers
