# Supplementary material for: Phosphoproteomic analysis reveals plant DNA damage signalling pathways with a functional role for histone H2AX phosphorylation in plant growth under genotoxic stress
Source: Plant J. 2019 Sep 10;100(5):1007–21. doi: 10.1111/tpj.14495 (PMC6900162; doi:10.1111/tpj.14495)
Supplement: Supplementary file 4 — Figure S4. Analysis of phosphosites. [file TPJ-100-1007-s004.pdf]

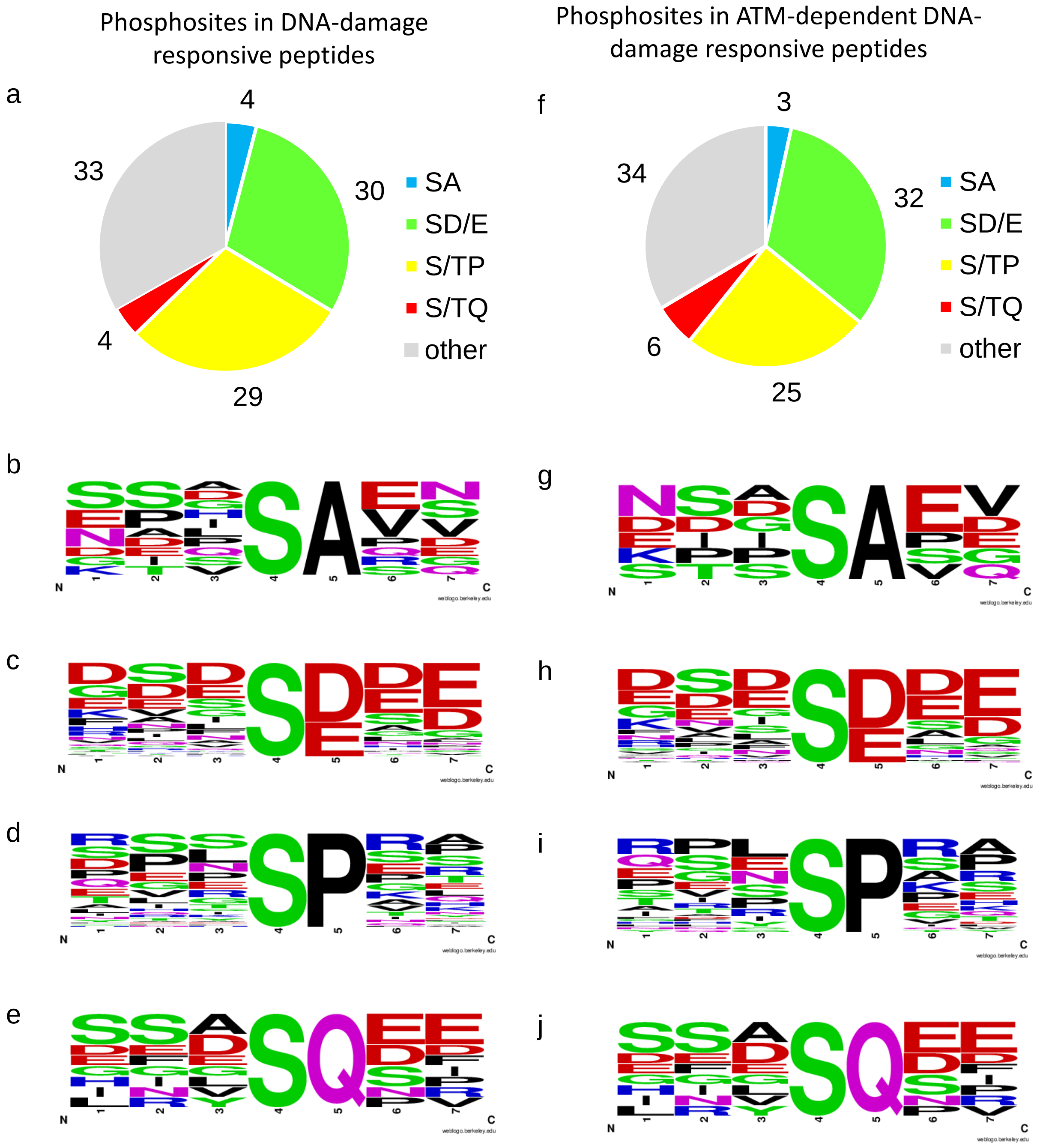
Phosphosites in DNA-damage responsive peptides
Phosphosites in ATM-dependent DNA-damage responsive peptides
a
4
30
29
4
33
SA
SD/E
S/TP
S/TQ
other
f
3
32
25
6
34
SA
SD/E
S/TP
S/TQ
other
b
c
d
e
g
h
i
j
